# The Health Effects of Motorization

**DOI:** 10.1371/journal.pmed.1001458

**Published:** 2013-06-11

**Authors:** Kavi Bhalla

**Affiliations:** Johns Hopkins International Injury Research Unit, Department of International Health, Johns Hopkins Bloomberg School of Public Health, Baltimore, Maryland, United States of America

## Abstract

Kavi Bhalla provides a perspective on new research by Christopher Millett and colleagues that reports rural dwellers who migrate to cities have worse cardiovascular outcomes, partly due to a shift to private motor vehicles and reduced physical activity.

*Please see later in the article for the Editors' Summary*

**Linked Research Article**

This Perspective discusses the following new study published in *PLOS Medicine*:

Millett C, Agrawal S, Sullivan R, Vaz M, Kurpad A, et al. (2013) Associations between Active Travel to Work and Overweight, Hypertension, and Diabetes in India: A Cross-sectional Study. PLoS Med 10(6): e1001459. doi:10.1371/journal.pmed.1001459

## The Rise of Non-communicable Diseases and Their Underlying Driving Forces

The world is undergoing a rapid transition in population health, away from mostly infectious disease in children to non-communicable diseases (NCDs) and injuries that affect adults [1],[2]. The global health transition is strongly tied to levels of industrial development in low- and middle-income countries. NCDs and injuries now comprise 94% of all deaths in China, 65% in India, and 34% in sub-Saharan Africa [2].

The study by Christopher Millett and colleagues, in this week's *PLOS Medicine* [3], paints a vivid picture of the sources of the transition to NCDs and injuries. Their paper highlights the shift in health risks that accompany migration from rural to urban settings, reporting that rural dwellers who migrate to cities have worse cardiovascular outcomes than their non-migrant siblings, partly due to a shift to private motor vehicles and reduced physical activity, especially reduced bicycling. While there is strong evidence from high-income countries linking physical inactivity to major NCDs, including coronary heart disease, type 2 diabetes, and breast and colon cancers [4], Millett and colleagues present important evidence from a rapidly developing economy. Of course, declines in physical activity are not the only risks faced by the urban migrants in their study. These migrants are also exposed to higher concentrations of urban air pollution and possibly higher risks for injuries in motor vehicle crashes.

Urbanization is one of a cluster of interconnected global forces shaping people's physical activity and their exposure to air pollution and injuries [5]. These megatrends include increases in personal motorization, population aging, food consumption, globalization of production, and electronic entertainment, among others. Public health practitioners are faced with the challenge of managing the production of health within this context. Furthermore, policy and planning decisions taken now will have long-term effects: whether countries in Asia and Africa build high-density mixed-use cities or choose low-density sprawl, for example, or, whether countries choose to motorize via motorcycles, cars, or public transit or choose transport through active modes. These decisions will determine levels of physical activity, vehicular emissions, and crash risks, and thus influence NCD and injury rates for future generations of urban dwellers.

## Promoting Active Transport around the World

Promoting active transport (including walking and biking) while simultaneously reducing private motor vehicles has emerged as a common vision for a wide range of civic actors because of the cardiovascular health benefits of physical activity and the potential of reducing vehicular emissions, which are an important risk factor for NCDs and a contributor to anthropogenic climate change [6]. These ideas of *sustainable transport* have support from urban planners, transport planners, and civil engineers, in addition to public health practitioners. They are also supported by the World Health Organization [7], the United Nations [8], the Intergovernmental Panel on Climate Change [9], and multi-lateral development banks [10].

Strategies for sustainable transport in regions around the world differ based on their development trajectory. For instance, in the United States, the development of highway infrastructure since the 1950s has shaped land use and resulted in sprawling cities geared towards private motor vehicles. Reversing this trend requires promoting dense urban designs that allow people to live close to places of work and recreation. In contrast, in many societies around the world, motorization has appeared recently and is driven by rising incomes in fairly small segments of society. Here public roads cater not only to cars but also to pedestrians, bicyclists, cycle rickshaws, and handcarts [11], symbolizing a relatively egalitarian mix of income and class groups. However, the growth in high-speed motor vehicles is squeezing out existing modes of active transport through a process driven by the growing risks that vehicles pose to non-motorized road-users and transport policies geared towards automobiles. A shifting of focus in transport policies to reprioritize active modes can improve the ability of all citizens to access desirable destinations.

## Active Transport Infrastructure That Is Safe, Secure, Pleasant, and Convenient

The determinants of active transport have important commonalities around the world, including that people tend not to walk, bike, or take public transport if they perceive these activities to be unsafe from injuries, insecure from crime, or unpleasant [12]. A growing body of literature on evaluation of active travel programs shows success with integrated approaches that include changes to infrastructure, active transport programs, supportive land use planning, advocacy and education, and restrictions on car use [13],[14].

Infrastructure is particularly important for safety of active travel. Vehicle speed reduction can result from changes to the built environment using speed bumps, curb extensions, chicanes, and roundabouts [15]. Where vehicle speed cannot be reduced sufficiently, unprotected road users need to be physically separated from vehicles through the provision of sidewalks, overpasses and underpasses, and separated bicycle lanes. In general, when pedestrians and bicycling facilities are provided, safety consistently improves.

Providing infrastructure for active transport results in more active users, especially women, who are more risk averse [16]. Similarly, urban designs that promote high visibility through good lighting and clear street views for residents and store owners make streets secure and encourage use by those particularly vulnerable to crime [17]. Infrastructure that makes biking, walking, and the use of public transport convenient also increases active transport [13], such as when public transit integrates secure and covered bicycle parking. High connectivity of roads, more and better quality sidewalks, and pedestrian shelters are important aspects of the built environment that have been shown to increase active transport [14].

There is growing evidence to suggest that concerted policy efforts can increase active travel. Motor vehicle use is already declining in some high-income countries [18], including the US. Although bike use in China, once known as the “Kingdom of Bicycles,” has declined substantially over the last three decades, there has seen a recent shift in bike policy in cities. China now has more than 39 bike share systems, the largest number of any country in the world. Similarly, after a long period of auto-centered transport policy in India, the recent national urban transport policy focuses on sustainable transport for all road users [11]. Promoting such policies that reverse growth of private motor vehicles and encourage active travel is critical to ensuring that we leave the next generation with a livable planet.

## Abbreviations

NCD: non-communicable disease
